# The influence of MMP-14, TIMP-2 and MMP-2 expression on breast cancer prognosis

**DOI:** 10.1186/bcr1503

**Published:** 2006-06-15

**Authors:** Bernard Têtu, Jacques Brisson, Chang Shu Wang, Hélène Lapointe, Geneviève Beaudry, Caty Blanchette, Dominique Trudel

**Affiliations:** 1 Department of Pathology, Laval University, Québec, Canada; 2 Centre de Recherche en Cancérologie, Laval University, Québec, Canada; 3 Unité de Recherche en Santé des Populations, Laval University, Québec, Canada

## Abstract

**Introduction:**

Matrix metalloproteinase (MMP)-2 is very active at degrading extracellular matrix. It is under the influence of an activator, membrane type 1 MMP (MMP-14), and the tissue inhibitor of metalloproteases (TIMP)-2. We hypothesized that the individual expression of these three markers or their balance may help to predict breast cancer prognosis.

**Methods:**

MMP-2, MMP-14 and TIMP-2 expression has been evaluated by 35S mRNA *in situ *hybridization on paraffin material of 539 breast cancers without distant metastasis at diagnosis and with a median follow-up of 9.2 years.

**Results:**

MMP-2 and MMP-14 mRNA was detected primarily in reactive stromal cells whereas TIMP-2 mRNA was expressed by both stromal and cancer cells. Of the three molecules, an adjusted Cox model revealed that high MMP-14 mRNA (≥ 10% cells) alone predicted a significantly shorter overall survival (*p *= 0.031) when adjusted for clinical factors (tumor size and number of involved lymph nodes). Prognostic significance was lost when further adjusted for Her-2/neu and urokinase-type plasminogen activator (*p *= 0.284). Furthermore, when all three components were analyzed together, the survival was worst for patients with high MMP-2/high MMP-14/low TIMP-2 (5 year survival = 60%) and best with low MMP-2/low MMP-14/high TIMP-2 (5 year survival = 74%), but the difference did not reach statistical significance (*p *= 0.3285).

**Conclusion:**

Of the MMP-14/TIMP-2/MMP-2 complex, MMP-14 was the factor most significantly associated with the outcome of breast cancer and was an independent factor of poor overall survival when adjusted for clinical prognostic factors, but not for certain ancillary markers.

## Introduction

In breast cancer, matrix metalloproteinase (MMP)-2 is a protease produced essentially by stromal cells. *In vitro *studies have clearly demonstrated that it degrades molecules that are abundant in the extracellular matrix (ECM) [1]. MMP-2 is also one of the major targets of recently developed synthetic MMP inhibitors [2,3]. However, recent literature demonstrates that the mechanism of action of MMP-2 is complex and that other molecules modulate its activity [4,5].

MMP-2 is secreted in an inactive pro-enzymatic form and, unlike other MMPs, its activity is modulated by tissue inhibitor of metalloproteases (TIMP)-2 [6] and the membrane type 1 MMP (MMP-14) [7]. Using zymography, breast cancers were found to express higher levels of activated MMP-2 than benign lesions [8,9] and *in vitro *studies showed that activated MMP-2 only is associated with an aggressive potential in breast cancer cell lines [10].

Animal studies showed that carcinoma cell lines transfected with MMP-14 produced higher levels of active MMP-2 and developed more lung metastases compared to parent tumor cells [11]. TIMP-2 inhibits most if not all activated MMPs and was shown to form a complex specifically with pro-MMP-2 [12]. However, the role of TIMP-2 is ambiguous since, in addition to its inhibitory effect, it is a main player in the MMP-2 activation cascade [13]. However, clinical studies on breast cancer are rare, limited in size and do not address the potential interaction of all three factors (MMP-2, MMP-14 and TIMP-2).

This prompted us to test the hypothesis that the individual expression or the balance between MMP-2, MMP-14 and TIMP-2 expression may help to predict breast cancer prognosis.

## Materials and methods

### Population

The patients included in this study had node-positive or node-negative disease proven by histological examination of axillary lymph nodes, but with absence of distant metastases at diagnosis. The surgery for breast carcinoma was performed between 1 January 1980 and 31 December 1986. Clinical information was obtained from the patients' charts by experienced research nurses. The tumor size, and number of examined and positive lymph nodes were obtained from the pathology reports. The histological types along with the histological and nuclear grades were reassessed by one of us (BT). The project has been approved by the Laval University Ethical Review board.

### *In situ *hybridization

MMP-2, MMP-14 and TIMP-2 expression was analyzed by *in situ *hybridization. The technique used was a modification of the method of Wolf and colleagues [14] as described before [15]. S^35 ^labeled antisense RNA probes were used. Plasmids were kindly provided by the late Prof. Paul Basset, IGBMC, Illkirch, France (MMP-2 and MMP-14) and the late Dr Anna Kossakowska, Calgary, Alberta (TIMP-2). cDNA inserts were prepared from breast cancer cDNA libraries, subcloned in pBluescript II (MMP-2 and MMP-14) and pBS KS- (TIMP-2) vectors and were used as templates for *in vitro *transcription to generate sense and anti-sense probes. The 2,124 base-pair (bp) MMP-2 cDNA was subcloned in the *Eco*RI site and extended from nucleotide 26 to 2,150; the 1,159 bp MMP-14 cDNA was subcloned in the *Eco*R1 site and extended from nucleotide 454 to 1,613; and the 210 bp TIMP-2 cDNA was subcloned in the *Hin*dIII/*Bam*H1 site. Reduction of the probe length was achieved by partial alkaline hydrolysis. The quality of RNA preservation has been assessed with the use of antisense RNA probes for β-actin and negative controls were obtained with the use of sense probes. *In situ *hybridization for MMP-11 and urokinase-type plasminogen activator (uPA) were performed on all cases as previously described [15].

### Immunohistochemistry

In addition to *in situ *hybridization, an immunohistochemical study was performed using the avidin-biotin complex (ABC) method as described before [15]. Studies were performed using primary antibodies to cathepsin D (Novocastra, Newcastle, UK; dilution, 1/200), p53 (Signet Labs, Dedham, MA, USA; ID labs, London, Ontario, Canada; dilution, 1/50), the heat-shock protein of 27 kDa (HSP-27, Hu27, Dr Jacques Landry, Hôtel-Dieu de Québec, Canada; dilution, 1/200), and HER-2/neu (Triton Biosciences, Alameda, CA, USA; dilution, 1/15). For each antibody, positivity was defined by the presence of at least 10% of cells expressing the marker. P53 expression was nuclear, HER-2/neu was membranous and HSP-27 was cytoplasmic.

### Flow cytometry

DNA flow cytometric analysis was performed in all cases on formalin fixed-paraffin embedded tissues using the method described by Dressler and colleagues [16]. Ploidy and S-phase fraction were determined on single parameter histograms with the use of ModFit^® ^(Verity Software House Inc., Topsham, ME, USA). Debris were excluded with the use of the 'Single Cut' algorithm. G0, G1 and G2 M were defined from a Gaussian curve while S-phase fraction was evaluated with the trapezoid model. All cases with a coefficient of variation exceeding 8% were excluded; the coefficient of variation averaged 6.1% (range 2.5% to 8.0%).

### Hormone receptors

Estrogen and progesterone receptor measurement was obtained from the patient charts. In the early 1980s, analyses were performed using the hydroxylapatite method [17]. Positivity was defined by hormone levels above 10 fmol/mg proteins.

### Interpretation

The light microscopic interpretation of *in situ *hybridization was done by two of us (BT, DT) without knowledge of the clinical information. The scoring was assessed separately on cancer and stromal cells using a semi-quantitative scale similar to that used for immunohistochemistry. For each cell compartment, the percentage of cancer or stromal cells expressing the marker (0%, <10%, 10% to 50%, >50%) was evaluated on the whole tumor surface of one representative section.

### Statistical analysis

Distant-metastasis-free and overall survival (OS) curves were obtained for each protease according to Kaplan and Meier [18]. The difference between the curves was assessed using the log-rank test [19]. Correlation between protease expression and other recognized prognostic variables in breast cancer was determined by the Chi-square test [19]. Distant-metastasis-free and OS curves were generated using 10% as the cutoff point between those expressing low (negative) or high (positive) protease levels by stromal cells, as defined before [15,20]. The Cox proportional hazard model was used to evaluate the relationship between the expression of each protease, or a combination of more than one protease, and recurrence or death, adjusting for other known or suspected prognostic factors [21]. Hazard ratios for the occurrence of distant metastases or death of patients with any of the above combinations were obtained by univariate and multivariate analyses.

## Results

In our tumor bank, information for MMP-2 was available from 610 breast cancers, from 544 for MMP-14 and from 549 cases for TIMP-2. Results for all three molecules were available from 539 cases and all analyses have been performed on this group of patients. The patients' ages ranged from 29 to 72 years (average, 57.3). Of these patients, 190 (35.3%) were node-negative, 340 (63.1%) had node invasion, and the node status was unknown in 9 (1.6%). At last follow-up, 258 (47.9%) were alive and the follow-up of surviving patients ranged from 5.2 to 14.6 years (average, 9.5 years); 21 (3.9%) were lost to follow-up.

**Figure 1:**
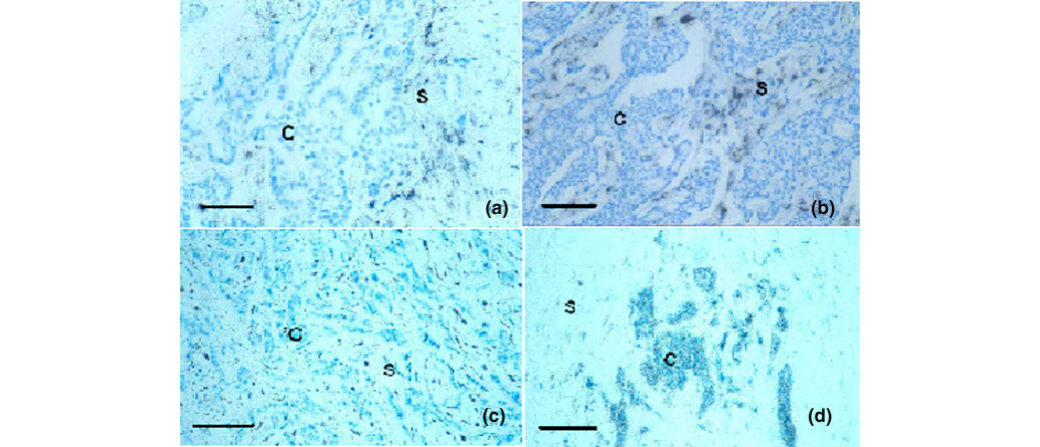
Expression of matrix metalloproteinase (MMP)-2, MMP-14 and tissue inhibitor of metalloproteases (TIMP)-2 in stromal and cancer cells. **(a) **MMP-2 expression by stromal cells (*in situ *hybridization, × 200); **(b) **MMP-14 expression by stromal cells (*in situ *hybridization, × 200); **(c) **TIMP-2 expression by stromal cells (*in situ *hybridization, × 200); **(d) **TIMP-2 expression by cancer cells (*in situ *hybridization, × 200). Scale bar = 200 microns. C, cancer cells; S, stromal cells.

In this study, stromal cells expressed high MMP-2 (Figure 1a) in 263 (48.8%) cases, high MMP-14 (Figure 1b) in 164 (30.4%) cases and high TIMP-2 (Figure 1c) in 200 (37.1%) cases. Furthermore, high TIMP-2 expression by cancer cells (Figure 1d) was present in 225 (41.7%) cases while cancer cells expressed MMP-14 in 4 (0.7%) cases and none expressed MMP-2.

MMP-14 expression by stromal cells was significantly associated with a younger age, lymph node involvement, a ductal histology, HER-2/neu overexpression, and cathepsin D, MMP-11 (stromelysin-3) and uPA expression by stromal cells (Table 1). MMP-2 expression by stromal cells was associated with lymph node involvement, ductal histology, HER-2/neu and HSP-27 overexpression as well as cathepsin D, MMP-11 and uPA expression by stromal cells. TIMP-2 expression by stromal cells was associated with a ductal histology, and expression of HSP-27 and uPA. TIMP-2 expression by cancer cells was associated with peritumoral lymphovascular invasion, and expression of HSP-27, cathepsin D and uPA by stromal cells.

**Figure 2:**
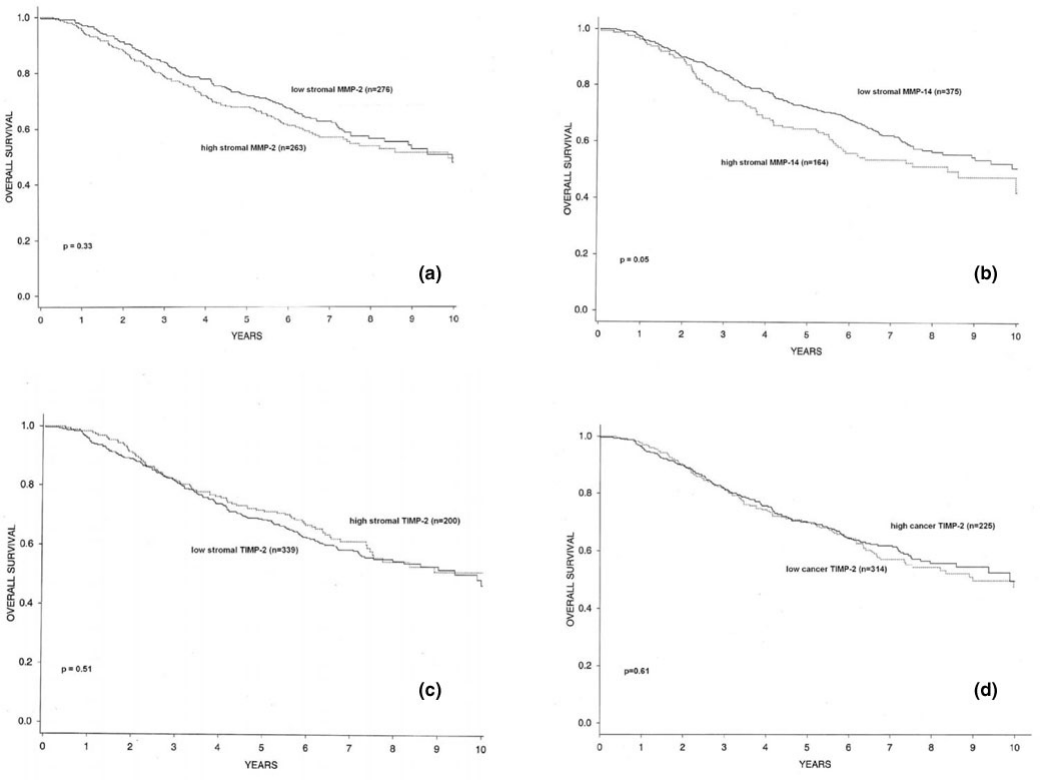
Overall survival for matrix metalloproteinase (MMP)-2, MMP-14 and tissue inhibitor of metalloproteases (TIMP)-2 expression by stromal and cancer cells. Overall survival curves for: **(a) **matrix metalloproteinase (MMP)-2 expressed by stromal cells; **(b) **MMP-14 expressed by stromal cells; **(c) **tissue inhibitor of metalloproteases (TIMP)-2 expressed by stromal cells; and **(d) **TIMP-2 expressed by cancer cells (*p *values obtained by the log-rank test).

Figure 2 shows the OS curves for each of the three proteases. MMP-2 and MMP-14 expression by cancer cells was not evaluated because too few cases expressed those markers. No difference in OS was found between high and low MMP-2 and between high and low stromal and cancer TIMP-2 expression. However, high MMP-14 expression by stromal cells predicted a shorter survival (*p* = 0.05). In fact, the 5 year survival reached 72.2% for patients whose tumors had low MMP-14 levels as opposed to 64.6% for those with high MMP-14 levels. By multivariate analysis, using the Cox model adjusting for tumor size and number of involved lymph nodes, high MMP-14 expression was clearly a significant factor of poor survival (Table 2). The statistical significance was lost when HER-2/neu and uPA were included in the model. A trend was found for the association of MMP-14 expression and metastasis-free survival (*p *= 0.08). Indeed, the 5 year metastasis-free survival was 64.0% for patients with low MMP-14 as opposed to 54.3% for those with high MMP-14.

**Figure 3:**
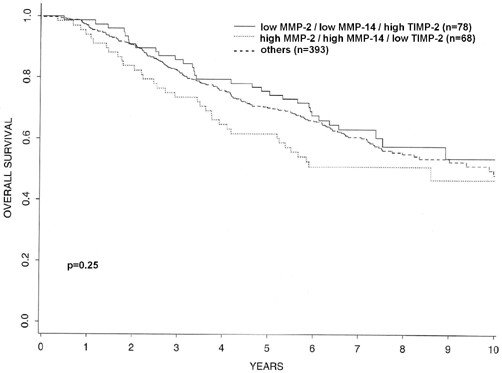
Overall survival curves for: matrix metalloproteinase (MMP)-2, MMP-14 and stromal tissue inhibitor of metalloproteases (TIMP)-2 (*p *values obtained by the log-rank test).

Figure 3 shows the overall survival curve considering the balance between MMP-2, MMP-14 and TIMP-2. Of the different combinations, OS was worst for those patients with high stromal MMP-2/high stromal MMP-14/low stromal TIMP-2 (60.3% survival at 5 years) and best with low stromal MMP-2/low stromal MMP-14/high stromal TIMP-2 (74.4% survival at 5 years). This difference did not, however, reach significance (*p *= 0.25).

## Discussion

This study confirms the complexity of the role of proteases on breast cancer prognosis. However, the prognostic influence of MMP-2 expression is not clear in the literature. Talvensaari-Mattila and colleagues [22], Sivula and colleagues [23] and Pacheco and colleagues [24] are among the few investigators who demonstrated a significant association between high MMP-2 mRNA or protein expression levels and a poor outcome. In our study of close to 600 cases, MMP-2 overexpression predicted a 15% greater risk of recurrence [15] but the association of MMP-2 expression with prognosis was not statistically significant. Other studies have failed to relate MMP-2 expression to a poorer prognosis [2,15,25]. These data are consistent with the complexity of the biology of MMP-2. In our study, they may also be explained in part by the fact that *in situ *hybridization cannot differentiate between the active and inactive forms.

In our study, when each factor was taken separately, and after adjustment for clinical prognostic factors, only MMP-14 was a significant factor of survival, suggesting that MMP-14 may be one of the key steps in tumor invasion and metastasis. However, the prognostic significance of MMP-14 was not independent of HER-2/neu and uPA. This finding can be explained by the strong regulatory interaction between HER-2/neu, uPA and MMPs [26]. Indeed, HER-2/neu was found to induce uPA expression and to directly up-regulate MMP expression via transcription factor-binding sites [26].

However, the association of MMP-14 but not MMP-2 expression with survival may be explained by the fact that MMP-14 has many substrates other than MMP-2. Indeed, *in vitro *and clinical studies support the major role played by MMP-14 on ECM remodeling. MMP-14 acts either directly by degrading ECM components such as type III collagen or indirectly by activating pro-MMP-2 and also by inducing highly vascularized tumors through vascular endothelial growth factor (VEGF) up-regulation [27-30]. MMP-14 also induces functional activation of the integrin αVβ3, which binds to MMP-2 and increases vitronectin-mediated adhesion and migration of MCF7 cells [31]. Among prior clinical studies, high MMP-14 mRNA expression was an independent factor of both tumor invasion and lymph node metastasis in carcinoma of stomach [32], lung [33] and cervix [34]. In breast cancer, highest expression of MMP-14 by RT-PCR was found in cases with lymph node metastases [35,36], poor clinical stage and larger tumor size [36].

In our study, MMP-14 mRNA was located in reactive stromal cells close to cancer cells. This is consistent with data from the literature [37] that identified MMP-14 mRNA within myofibroblasts [38]. However, the location of MMP-14 in breast tissue is debated and others found MMP-14 mRNA in cancer cells [39]. Using immunohistochemistry, MMP-14 was located within either stromal and/or cancer cells [40-43].

In our study, TIMP-2 did not provide independent prognostic information. In the literature on breast cancer, the role of TIMP-2 was controversial. This may explain contradictory results in which patients with higher TIMP-2 expression either experienced low cancer recurrence/progression [44,45] or a poor prognosis [25,46-49], emphasizing the overall activating or inhibitory role of TIMP-2. These contradictory findings may be explained by the versatile role of TIMP-2 [12]. Indeed, TIMP-2 increases growth rates of murine, bovine and human cells [50], but also inhibits tumor growth and angiogenesis in melanoma B16F10 cells [51], attenuates migration of MDA-MB23 breast cancer cells through a bone marrow fibroblast monolayer [52] and abolishes the tumor-promoting effect of fibroblasts on MCF7 cells injected with matrigel in nude mice [53].

The relationship of survival with the combination of MMP-2, MMP-14 and TIMP-2, although not statistically significant, showed that survival was worst for those patients with high MMP-2/high MMP-14/low TIMP-2 and best with low MMP-2/low MMP-14/high TIMP-2. This lack of statistical significance may be explained by the sample size, which may be too small to reach significance, or by the versatile role of TIMP-2, which may be either a favorable or an unfavorable factor. Different combinations of proteases and protease inhibitors or activators have been investigated in the literature but clinical studies are limited and involve few patients. Clinical studies suggest that TIMP-2, or the ratio of MMP-2 and TIMP-2 expression, may play a critical role on cancer progression. For example, higher MMP-2/TIMP-2 ratios were associated with recurrences in patients with bladder [54] or uterine cervix [55] cancer. In a study on 14 patients, Onisto and colleagues [56] report that a high MMP-2/TIMP-2 ratio correlated with lymph node metastases in breast cancer while it predicted a better outcome in another study [25].

## Conclusion

Our data show that, of the three proteases studied, MMP-14 was most strongly associated with breast cancer prognosis but was not independent of HER-2/neu or uPA, which may limit its usefulness as a prognostic marker. When all three proteases are studied in combination, a tendency was found for tumors with high MMP-2, high MMP-14 and low TIMP-2 expression to predict a poor prognosis but the results did not reach significance.

## Abbreviations

bp = base-pair; ECM = extracellular matrix; HSP = heat-shock protein; MMP = matrix metalloproteinase; OS = overall survival; TIMP = tissue inhibitor of metalloproteases; uPA = urokinase-type plasminogen activator.

## Competing interests

The authors declare that they have no competing interests.

## Authors' contributions

BT, JB, CSW, and DT participated in the design of the study. CSW, HL and GB developed and prepared all *in situ *hybridization analyses. BT and DT interpreted all *in situ *hybridization slides. JB supervised the statistical analysis, which was carried out by CB. All authors participated to the discussion on the significance of the results, the drafting of the manuscript and approved the final version.

## Figures and Tables

**Table 1:** Association of high protease expression by stromal cells with other prognostic factors

Parameter	Cases	MMP-14			MMP-2			TIMP-2: stromal cells			TIMP-2: cancer cells		
		Number	Percentage Pos	*p *value	Number	Pecentage Pos	*p *value	Number	Percentage Pos	*p *value	Number	Percentage Pos	*p *value

Age years													
<50	160	59	36.9	0.034	85	53.1	0.19	58	36.3	0.79	59	36.9	0.14
≥50	379	105	27.7		178	47.0		142	37.5		166	43.8	
Tumor size cm													
<3	297	93	31.3	0.98	146	49.2	0.65	118	39.7	0.14	122	41.1	0.83
≥3	207	65	31.4		106	51.2		69	33.3		87	42.0	
Involved lymph nodes													
0	190	46	24.2		81	42.6		71	37.4		80	42.1	
1 to 3	166	61	36.7	0.036	93	56.0	0.041	69	41.6	0.29	75	45.2	0.52
>3	174	54	31.0		84	48.3		58	33.3		68	39.1	
Peritumoral lymphovascular invasion													
Yes	143	50	35.0	0.15	76	53.1	0.097	50	35.0	0.15	64	44.8	0.027
No	75	19	25.3		31	41.3		19	25.3		22	29.3	
Histological grade													
I	54	18	33.3		25	46.3		19	35.2		23	42.6	
II	277	81	29.2	0.69	133	48.0	0.83	109	39.4	0.59	119	42.9	0.82
III	197	64	32.5		99	50.3		69	35.0		79	40.1	
Nuclear grade													
I	94	20	21.3		40	42.6		39	41.5		42	44.7	
II	307	98	31.9	0.066	158	51.5	0.27	105	34.2	0.22	128	41.7	0.79
III	127	45	35.4		59	46.5		53	41.7		51	40.2	
Histological type													
Invasive ductal carcinoma	470	156	33.2	0.0006	238	50.6	0.021	164	34.9	0.003	28	43.1	0.83
Others	65	8	12.3		23	35.4		35	53.8		196	41.7	
DNA ploidy													
Diploid	143	37	25.9	0.09	64	44.8	0.18	58	40.6	0.59	60	42.0	0.99
Non-diploid	332	112	33.7		171	51.5		126	38.0		139	41.9	
Estrogen receptors													
Negative	143	53	37.1	0.055	76	53.1	0.22	59	41.3	0.25	61	42.7	0.89
Positive	374	106	28.3		176	47.1		134	35.8		157	42.0	
Progesterone receptors													
Negative	219	74	33.8	0.21	109	49.8	0.72	88	40.2	0.26	95	43.4	0.60
Positive	297	85	28.6		143	48.1		105	35.4		122	41.1	
HER-2/neu expression													
Negative	437	121	27.7	0.0042	204	46.7	0.042	158	36.2	0.34	179	41.0	0.45
Positive	102	43	42.2		59	57.8		42	41.2		46	45.1	
p53													
Negative	374	107	28.6	0.12	186	49.7	0.53	132	35.3	0.24	149	39.8	0.30
Positive	152	54	35.5		71	46.7		62	40.8		68	44.7	
HSP-27													
Negative	204	61	29.9	0.097	97	47.5	0.02	63	30.9	0.0024	70	34.3	0.0003
Positive	157	60	38.2		94	59.9		73	46.5		84	53.5	
Cathepsin D cancer cells													
Negative	324	94	29.0	0.29	151	46.6	0.21	111	34.3	0.071	127	39.2	0.102
Positive	207	69	33.3		87	52.2		87	42.0		96	46.4	
Cathepsin D stromal cells													
Negative	303	78	25.7	0.0035	130	42.9	0.0013	106	35.0	0.21	116	38.3	0.047
Positive	226	85	37.6		129	57.1		91	40.3		106	46.9	
MMP-11													
Negative	215	32	14.9	<0.0001	65	30.2	<0.0001	76	35.3	0.46	93	43.3	0.60
Positive	322	132	41.0		198	61.5		124	38.5		132	41.0	
MMP-2													
Negative	276	41	14.9	<0.0001	-	-		89	32.2	0.017	110	39.9	0.36
Positive	263	123	46.8		-	-		111	42.2		115	43.7	
uPA													
Negative	374	81	21.7	<0.0001	147	39.3	<0.0001	127	34.0	0.016	143	38.2	0.017
Positive	160	81	50.6		115	71.9		72	45.4		79	49.4	

HSP, heat-shock protein; MMP, matrix metalloproteinase; Pos, positive; TIMP, tissue inhibitor of metalloproteases; uPA, urokinase-type plasminogen activator.

**Table 2:** Hazard ratios for MMP-14, MMP-2, TIMP-2 and combination of MMP-14 MMP-2 TIMP-2 on breast cancer overall survival

	Univariate			Multivariate^a^			Multivariate^b^		
	HR	95% CI	*p *value	HR	95% CI	*p *value	HR	95% CI	*p *value

MMP-14	1.31	0.99–1.72	0.05	1.36	1.03–1.81	0.031	1.18	0.87–1.59	0.28
MMP-2	1.14	0.88–1.47	0.33	1.14	0.87–1.49	0.34	0.98	0.73–1.31	0.87
TIMP-2: stromal cells	0.91	0.69–1.19	0.51	1.04	0.78–1.38	0.79	0.89	0.66–1.19	0.43
TIMP-2: cancer cells	1.07	0.83–1.39	0.61	1.09	0.83–1.43	0.53	1.02	0.77–1.34	0.91
MMP-14-/MMP-2-/TIMP-2+^c^	1.0			1.0					
MMP-14+/MMP-2+/TIMP-2-	1.46	0.89–2.38	0.13	1.29	0.77–2.16	0.33			
Other categories	1.11	0.76–1.63	0.59	1.03	0.68–1.55	0.90			

^a^Cox model adjusting for tumor size and number of involved lymph nodes. ^b^Cox model adjusting for tumor size, number of involved lymph nodes, HER-2/neu and urokinase plasminogen activator. ^c^TIMP-2 by stromal cells. CI, confidence interval; HR, hazard ratio; MMP, matrix metalloproteinase; TIMP, tissue inhibitor of metalloproteases.
